# Adaptation and validation of the Children’s Surgical Assessment Tool for Rwandan district hospitals

**DOI:** 10.1080/16549716.2023.2297870

**Published:** 2024-01-09

**Authors:** Sarah Nuss, Jean Paul Majyambere, Edmond Ntaganda, Callum Forbes, Jonathan Nkurunziza, Carol Mugabo, Vincent Cubaka, Bethany Hedt-Gauthier

**Affiliations:** a Program in Global Surgery and Social Change, Harvard Medical School, Boston, MA, USA; b Department of Surgery, Partners in Health Rwanda/Inshuti Mu Buzima, Butaro, Rwanda; c Department of Pediatric Surgery, Centre Hospitalier Universitaire de Kigali, Kigali, Rwanda; d Center for Equity in Global Surgery, University of Global Health Equity, Butaro, Rwanda; e Department of Global Health and Social Medicine, Harvard Medical School, Boston, MA, USA

**Keywords:** Global surgery, pediatric surgery, district hospital, rural, readiness assessment

## Abstract

**Background/Aims:**

Paediatric surgical care is a critical component of child health and basic universal health coverage and therefore should be included in comprehensive evaluations of surgical capacity. This study adapted and validated the Children’s Surgical Assessment Tool (CSAT), a tool developed for district and tertiary hospitals in Nigeria to evaluate hospital infrastructure, workforce, service delivery, financing, and training capacity for paediatric surgery, for use in district hospitals in Rwanda.

**Methods:**

We used a three-round modified Delphi process to adapt the CSAT to the Rwandan context. An expert panel of surgeons, anaesthesiologists, paediatricians, and health systems strengthening experts were invited to participate based on their experience with paediatric surgical or anaesthetic care at district hospitals or with health systems strengthening in the Rwandan context. We used the Content Validity Index to validate the final tool.

**Results:**

The adapted tool had a final score of 0.84 on the Content Validity Index, indicating a high level of agreement among the expert panel. The final tool comprised 171 items across five domains: facility characteristics, service delivery, workforce, financing, and training/research.

**Conclusion:**

The adapted CSAT is appropriate for use in district hospitals in Rwanda to evaluate the capacity for paediatric surgery. This study provides a framework for adapting and validating a comprehensive paediatric surgical assessment tool to local contexts in LMICs and used in similar settings in sub-Saharan Africa.

## Introduction

Surgical care is an essential component of child health, yet it is often overlooked. Lack of access to surgical care disproportionately affects low- and middle-income countries (LMICs), where children make up to 42% of the population, compared to 25% globally [1]. Recent estimates suggest that 61% of surgically treatable deaths in children under 20 years-old occur in LMICs [2]. Despite this, only an estimated 8% of the children in LMICs have access to surgical care [3]. Developing baseline metrics for paediatric surgical capacity is an important first step in understanding the unique operative, perioperative, and anaesthetic requirements for children that should be considered in efforts to scale up surgical services in LMICs.

To date, there are limited tools to assess paediatric surgery capacity in LMICs. The Pedi-PIPES tool (Paediatric Infrastructure, Process, Outcome and Cost Evaluation Score) is an example of one such tool. While there are strengths to this tool such as the incorporation of patient and family experience, overall it is limited in scope with a primary emphasis on quality and cost-effectiveness, and lack of emphasis on training, education, or adequate focus on neonatal conditions [4]. Various surgical subspecialties have also begun evaluating paediatric capacity through the development of specialty-specific paediatric Bellwether procedures, or essential procedures to be used as a proxy for access to paediatric subspecialty surgical care [5]. However, these lists are specialty specific and only capture a small subset of surgical conditions that may not reflect the full burden of surgical disease in children.

More recently, the Children’s Surgical Assessment Tool (CSAT) was developed to assess and guide strengthening of surgical systems based on currently available guidelines. The CSAT aims to identify gaps in facility infrastructure, service delivery, workforce, information management, and financing and has the potential to be adapted to the local context [6]. Compared to tools such as the Pedi-PIPES and paediatric Bellwether lists, the CSAT is a more comprehensive tool that covers a broader range of domains [7].

None of the existing tools comprehensively measure resources, outcomes, accessibility, and training [7]. The Global Assessment of Pediatric Surgery (GAPS) capacity assessment tool is currently being developed to fill this gap. However, this tool is being designed for paediatric surgical centres. It remains unclear whether this tool will be appropriate for use at district hospitals, which often serve as the primary entry point to surgical care for patients in LMICs. Given the different levels of infrastructure and training available at hospitals, the type of safe and appropriate surgical care differs between a first-level, district hospital and a tertiary referral hospital, and should be evaluated accordingly.

While the CSAT was developed in Nigeria with the potential to be used at a district hospital, the tool did not undergo validation in that setting. Furthermore, local regulations and guidelines around requirements for paediatric anaesthetic and operative care vary by country, leading to differing levels of appropriateness for some of the items listed on the original CSAT. The Global Initiative for Children’s Surgery (GICS) recently published an ‘Optimal Resources for Children’s Surgery (OReCS)’ consensus guideline for the optimal provision of paediatric surgical care, which provides recommendations for the infrastructure, training, and equipment required to provide essential paediatric surgical and anaesthetic care to children at each hospital level [8]. These guidelines are the first multi-national guidelines to provide hospital-level specific recommendations for paediatric surgical care.

In Rwanda and other LMICs, district hospitals are an important target for scaling up surgical services. One analysis showed that scaling up surgical care at district hospitals in LMICs could avert up to 22% of the surgical deaths at the district hospital level [2]. Baseline surgical capacity evaluations should be tailored to reflect the appropriate resources and training for a first-level hospital to ensure efforts to scale surgical capacity are done comprehensively, safely, and sustainably. Increasing access to surgical services at the district level is particularly important for children in Rwanda, with a significant burden of surgically treatable conditions found specifically in rural communities [9–11]. The goal of this study was to use the OReCS guidelines for first-level hospitals and expert consensus to adapt and validate the CSAT for use at district hospitals in Rwanda. In addition to a starting list for district hospitals in other sub-Saharan African countries, this paper outlines a framework for local adaptation of CSAT for other contexts.

## Methods

### Study setting

The healthcare system in Rwanda is divided into four tiers: community health workers who provide basic healthcare services and education in communities, health centres which provide primary care, district hospitals that provide more specialised care including surgical care, and referral hospitals which offer the highest level of specialised care. There are eight national referral hospitals, including three teaching hospitals (Centre Hospitalier Universitaire Kigali (CHUK) and Rwanda Military Hospital (RMH) in the capital, Kigali, and Centre Hospitalier Universitaire Butare (CHUB) in the Southern Province) and a tertiary hospital in Kigali (King Faisal Hospital) [12]. Recently three district hospitals were upgraded to referral hospitals and four district hospitals were upgraded to provincial hospitals to decrease the pressure of demand for services in the national referral hospitals. There is a national-based health insurance scheme which provides health insurance to over 95% of the population.

In 2017 Rwanda took a significant step towards Universal Health Coverage by developing a National Surgical, Anesthetic, and Obstetric Plans (NSOAPs). The Rwandan Ministry of Health conducted an evaluation of their NSOAP and found that the total surgical volume per population in Rwanda including cases performed in tertiary centres was calculated to be 786 procedures per 100,000 population, which is much less than the 5,000 procedures per 100,000 population recommendation [13]. A retrospective chart review found a total of 1274 children were operated at CHUK and CHUB hospitals from 2013 to 2014 [14]. The most common surgical conditions among paediatric patients were trauma and burn (36.58%), congenital anomaly (23.39%), and surgical infections (14.76%) [14]. The total surgery, anaesthesia, and obstetric (SAO) provider density is 1.006 per 100,000 population, which is much below the target 20–40 SAO providers per 100,000 population, and many of these specialists are concentrated in tertiary centres rather than DHs [13].

One of the major goals of the Rwanda NSOAP has been to increase district hospital surgical capacity by prioritising increasing the Ministry of Health budget allocated to SOA care at DHs. There are 36 district hospitals and four provincial hospitals in Rwanda with variable ability to provide surgical care. The 2017 Rwanda NSOAP evaluation found that across the district hospitals there were an average of 1,703 surgical cases performed per hospital. There are just 25 surgical, anaesthetic, and obstetrics (SAO) providers across the 36 district hospitals.

While Rwanda's NSOAP acknowledges paediatric surgical care as a subspecialty available at tertiary referral centres, there is no specific provision for it at the district hospital level. This presents a crucial gap that needs to be addressed, given that most children access healthcare services through DHs. The shortage of paediatric surgeons in the country (three fellowship-trained paediatric surgeons), coupled with a high volume of referrals from DHs, can lead to delays in care, resulting in potentially preventable morbidity and mortality. In Rwanda, individuals less than 21 years are twice as likely to have an untreated surgical condition [9]. Yet, children in rural Rwanda are much more likely to experience delay in referrals compared to adults with traumatic injuries [15].

The assessment tool was adapted for use at the Ministry of Health district hospitals in rural Rwanda: Rwinkwavu District Hospital and Kirehe District Hospital in Eastern Province, and Butaro District Hospital in Northern Province. These are government-run facilities supported by Partners In Health (PIH), a US-based non-governmental organisation that accompanies the Ministry of Health in Rwanda in strengthening health systems.

#### Study overview

As a first step, the study team developed a preliminary adaptation of CSAT based on the OReCS guidelines. After the preliminary adaptation was completed, an expert panel was invited to participate in a three-round modified Delphi adaptation to develop consensus on the tool. The final tool was validated using the Content Validity Index (CVI) [16].

#### Expert panel recruitment

Expert panellists were invited based on experience with paediatric surgery, paediatric anaesthesia, or paediatric operative care at a District Hospital, or health systems strengthening in the local context (i.e. East Africa). Experts were identified by purposive sampling and invited to participate by the study team. To ensure local representation, over 50% of panellists were to be Rwandan experts. The expert panellists did not receive financial compensation for their participation and are included as co-authors in this paper.

#### Modified delphi method

During the internal preliminary adaptation conducted by the study team, all items on the original CSAT were sorted into three categories (propose to keep, propose to remove, and require additional discussion) based on the OReCS guidelines for a district hospital level facility. In the first round, this proposed adaptation was presented to each member of the expert panel. Individual semi-structured in-person or virtual interviews were conducted with each expert panelist to review the preliminary adaptation based on their personal experience. Feedback was recorded through notes taken during the interview and stored online. After all interviews were complete, feedback from the panellists was aggregated, and expert panellists were invited to attend one of the three optional virtual expert group sessions to discuss the feedback in a group setting. Final feedback from individual interviews and the expert group virtual meetings were used to deem each item as ‘retained’ from the original tool, ‘removed,’ and new items added by the expert panel feedback were deemed ‘added by expert panel’ (Table 2) for the second-round adaptation of the tool.

In the second round, expert panellists were asked to rate the relevance of each item on the adapted tool on a 4-point Likert scale (1 = not relevant at all, 2 = somewhat relevant, 3 = quite relevant, 4 = highly relevant) through an online survey. Participants were also given the opportunity to provide additional feedback on each section. Items that received a score of one or two were deemed not relevant and items that received a score of three or four were deemed relevant. The proportion of expert panellist who deemed each item as relevant was calculated. Items that received an average score of >0.75 were kept in the tool, and items with a score <0.75 were subjected to a third round of expert review. Expert panellists were able to provide written feedback during Round 2 of the survey.

In the third round, expert panellists were asked to rate each item with a score <0.75 as necessary or not necessary. Items with >50% of the respondents deeming the item as necessary were included in the final tool.

#### Validation

We used a CVI to validate the tool [16]. Each item on the tool was given an item validity score (I-CVI). I-CVI scores were calculated based on the proportion of participants who deemed items that were included on the final tool relevant (proportion of items ranked 3 or 4 on second-round survey, or proportion of items ranked as necessary, if included on the third round). An average validity (S-CVI/Average) was calculated by averaging all I-CVI scores. In alignment with researcher recommendations for minimum acceptable S-CVI Score, a threshold of an S-CVI score ≥ 0.8 was used to establish validity of the tool [17].

## Results

Eighteen expert panellists contributed to the adaptation and validation process. Thirteen (72%) were from Rwanda. Table 1 provides breakdown of expert panellists by specialty and country of practice.

**Table 1.** Expert panellists.

		*N* = 18
Country		
	Rwanda	14 (77.8%)
	USA*	4 (22.2%)
Role		
	Pediatric Surgeon	6 (33.3%)
	Pediatric Anesthesiologist	5 (27.78%)
	General Surgeon	1 (5.56%)
	Surgical Sub-Specialty	2 (11.11%)
	Pediatrician	1 (5.56%)
	Health Systems Researcher	3 (16.67%)
Years Experience		
	1–3	3 (16.67%)
	3–5	3 (16.67%)
	5–10	4 (22.22%)
	>10	8 (44.45%)
Institutions Represented**		
	Centre Hospitalier Universitaire Kigali	8
	Rwanda Military Hospital	4
	King Faisal Hospital	1
	Butaro District Hospital	2
	Kirehe District Hospital	1
	University of Global Health Equity	3
	Partners in Health Rwanda	4
	Harvard Medical School	2
	Duke University Medical School	1
	University of Florida Medical School	1

*All panel members from the United States have at least 10 years of experience living and working in Rwanda.

**Several panelists represent and have worked for multiple institutions.

### Round 1

Sixteen of the (88.9%) semi-structured interviews were conducted with expert panellists. Thirteen (72%) participated in one of the three virtual group discussions. Forty-three new items were added to the tool for further discussion and group evaluation (Table 2). Based on expert panellist feedback, the survey was adapted to differentiate the ability to provide care to two different age groups: patients under 5 years old and patients 5–15 years old given the national regulations requiring a physician anaesthesiologist to be present for any procedure requiring general anaesthesia. To do this, 10 questions on the survey were duplicated and modified to ask about only children <5 years old. The corresponding original questions were modified to ask about children 5–15 years old.

**Table 2.** (a-f) CSAT delphi method adaptation results.

Item	Round 1	Round 2 Score	Round 3 Score	Included in Final Tool?
**a. FACILITY INFORMATION**				
Total number of pediatric inpatient admissions (>6 hours in non-emergency ward) for children < 5 in a year	Introduced by Expert Panel	0.94	N/A	Yes
Total number of pediatric ED visits for children < 5 in a year	Introduced by Expert Panel	0.88	N/A	Yes
Total number of pediatric outpatient visits for children < 5 in a year	Introduced by Expert Panel	0.88	N/A	Yes
Total number of children’s surgical admissions for children < 5 in a year	Introduced by Expert Panel	0.94	N/A	Yes
Total number of children’s surgical outpatients (visits) for children < 5 seen in a year	Introduced by Expert Panel	0.88	N/A	Yes
Total number of pediatric inpatient admissions (>6 hours in non-emergency ward) for children 5–15 years-old in a year	Retained from CSAT	0.88	N/A	Yes
Total number of pediatric ED visits for children 5–15 years-old in a year	Introduced by Expert Panel	0.71	0.94	Yes
Total number of pediatric outpatient visits for children 5–15 years-old in a year	Introduced by Expert Panel	0.71	0.76	Yes
Total number of children’s surgical admissions for children 5–15 years-old in a year	Retained from CSAT	0.76	N/A	Yes
Total number of children’s surgical outpatients (visits) for children 5–15 years-old seen in a year	Retained from CSAT	0.82	N/A	Yes
Total number of inpatient hospital beds dedicated to children’s surgery	Retained from CSAT	0.82	N/A	Yes
Does your hospital have a pediatric recovery room?	Introduced by Expert Panel	0.76	N/A	Yes
Total number of recovery room hospital beds dedicated to children’s surgery	Introduced by Expert Panel	0.82	N/A	Yes
Is oxygen available in the recovery room?	Introduced by Expert Panel	1.00	N/A	Yes
Total number of operating rooms in the hospital	Introduced by Expert Panel	0.88	N/A	Yes
Total number children’s operating rooms	Retained from CSAT	0.82	N/A	Yes
Total number of neonatal unit/special baby care unit beds	Retained from CSAT	1.00	N/A	Yes
Does your hospital have a post-operative ICU?	Introduced by Expert Panel	0.88	N/A	Yes
Total number of pediatric ICU/advanced care beds	Retained from CSAT	0.88	N/A	Yes
Total number of neonatal ICU beds	Retained from CSAT	0.82	N/A	Yes
Total number of functional pediatric ventilators in the ICU	Retained from CSAT	0.82	N/A	Yes
Total number of functional neonatal ventilators in ICU	Retained from CSAT	0.76	N/A	Yes
Total number of functional incubators or pediatric warmers	Modified	0.94	N/A	Yes
Total number of patient monitors in pediatric and neonatal ICU	Retained from CSAT	0.88	N/A	Yes
How many of these monitors include all of the following: EKG, blood pressure, pulse oximetry, temperature and capnography?	Introduced by Expert Panel	0.88	N/A	Yes
How many patients <5 years old per year do you refer to higher-level facilities for surgical interventions?	Introduced by Expert Panel	0.88	N/A	Yes
How many patients 5–15 years old per year do you refer to higher-level facilities for surgical interventions?	Retained from CSAT	0.88	N/A	Yes
What is the most common reason for referral of pediatric surgical patients <5 years old to higher levels of care?	Introduced by Expert Panel	0.94	N/A	Yes
What is the most common reason for referral of pediatric surgical patients 5–15 years old to higher levels of care?	Retained from CSAT	0.94	N/A	Yes
How far away is the nearest referral hospital (hours, by car)	Introduced by Expert Panel	0.94	N/A	Yes
**b. INFRASTRUCTURE**				
24-hour Emergency Unit able to receive pediatric patients	Retained from CSAT	1.00	N/A	Yes
Pediatric malnutrition feeding program	Introduced by Expert Panel	0.71	0.71	Yes
Pediatric dosing cognitive aid or guide	Introduced by Expert Panel	0.76	N/A	Yes
Does your hospital have a radiologist?	Retained from CSAT	0.82	N/A	Yes
Does your hospital have an anesthesiologist who can perform sedation?	Modified	0.94	N/A	Yes
Enteral contrast material for bowel evaluation (barium or gastrografin)	Retained from CSAT	0.69	0.50	Yes
Intravenous contrast material	Retained from CSAT	0.63	0.31	No
Echocardiogram	Retained from CSAT	0.80	N/A	Yes
Ultrasound	Introduced by Expert Panel	1.00	N/A	Yes
X-ray	Introduced by Expert Panel	1.00	N/A	Yes
Anatomic Pathology services	Retained from CSAT	0.63	0.56	Yes
Blood component transfusion	Retained from CSAT	0.94	N/A	Yes
**c. SERVICE DELIVERY**				
Suturing laceration	Retained from CSAT	0.94	N/A	Yes
Drainage of superficial abscess	Retained from CSAT	1.00	N/A	Yes
Wound debridement	Retained from CSAT	1.00	N/A	Yes
Biopsy (tumour)	Retained from CSAT	0.88	N/A	Yes
Male circumcision	Retained from CSAT	1.00	N/A	Yes
Management of non-displaced fractures	Retained from CSAT	1.00	N/A	Yes
Removal of foreign body from ear/nose	Retained from CSAT	1.00	N/A	Yes
Support for emergency airway obstruction	Modified	0.94	N/A	Yes
Eyelid surgery for trachoma	Retained from CSAT	0.73	0.47	No
Reduction of dislocation	Introduced by Expert Panel	0.94	N/A	Yes
Reduction and application of splint for non-displaced fractures	Introduced by Expert Panel	1.00	N/A	Yes
IV placement for neonates	Introduced by Expert Panel	0.94	N/A	Yes
Appendectomy	Retained from CSAT	0.88	N/A	Yes
Laparoscopy for typhoid peritonitis	Introduced by Expert Panel	0.56	0.38	No
Hernia/hydrocele repair	Introduced by Expert Panel	0.93	N/A	Yes
Non-operative reduction of intussusception	Introduced by Expert Panel	0.88	N/A	Yes
Operative reduction of intussusception	Modified	0.88	N/A	Yes
Bowel resection	Retained from CSAT	0.87	N/A	Yes
Temporizing measures for gastroschisis and omphalocele (cover and rehydration) prior to referral	Modified	0.94	N/A	Yes
Rectal biopsy	Retained from CSAT	0.56	0.69	Yes
Resection of solid abdominal masses	Retained from CSAT	0.63	0.73	Yes
Creation of intestinal stomas	Retained from CSAT	0.94	N/A	Yes
Closures of intestinal stomas	Retained from CSAT	0.81	N/A	Yes
Emergency ostomies for imperforate anus	Introduced by Expert Panel	0.81	N/A	Yes
Resuscitation for pyloric stenosis	Modified	0.94	N/A	Yes
Catheterization/suprapubic cystostomy	Retained from CSAT	1.00	N/A	Yes
Orchiopexy	Retained from CSAT	0.88	N/A	Yes
Repair of testicular or ovarian torsion	Retained from CSAT	0.88	N/A	Yes
Thyroidectomy (total or partial)	Retained from CSAT	0.38	0.44	No
Drainage of septic arthritis/osteomyelitis	Retained from CSAT	0.93	N/A	Yes
Repair of cleft lip and/or palate	Retained from CSAT	0.53	0.59	Yes
Central Lines	Removed	N/A	N/A	No
Emergency Surgical airway (cricothyroidotomy)	Modified	0.94	N/A	Yes
Emergency Tube thoracostomy	Modified	0.94	N/A	Yes
Trauma Laparotomy	Retained from CSAT	0.88	N/A	Yes
Conservative (non-operative) management for simple humeral fracture	Introduced by Expert Panel	0.88	N/A	Yes
Open reduction and internal fixation	Retained from CSAT	0.73	0.88	Yes
Placement of pediatric external fixator	Retained from CSAT	0.75	N/A	Yes
Emergency Escharotomy/fasciotomy	Modified	0.75	N/A	Yes
Contracture release	Modified	0.56	0.63	Yes
Amputations	Retained from CSAT	0.94	N/A	Yes
Skin grafting	Retained from CSAT	0.67	N/A	Yes
Emergency Burr hole	Modified	0.80	N/A	Yes
Emergency Craniotomy	Modified	0.80	N/A	Yes
Surgery for neonatal acute abdomen	Introduced by Expert Panel	0.81	N/A	Yes
Repair of club foot	Retained from CSAT	0.57	0.65	Yes
Placement and management of external ventricular drain	Modified	0.73	0.50	No
Definitive repair of non-complicated anorectal malformation or Hirschsprung’s Disease	Modified	0.63	0.50	No
Repair of esophageal atresia	Removed	N/A	N/A	No
Repair of intestinal atresia	Removed	N/A	N/A	No
Myelomeningocele reapir	Removed	N/A	N/A	No
Atrial/Ventricular Septal Defect Repair	Removed	N/A	N/A	No
Number of pediatric laparotomies (<5 y/o) performed last year	Introduced by Expert Panel	0.88	N/A	Yes
Number of pediatric laparotomies (5–15 y/o) performed last year	Retained from CSAT	0.88	N/A	Yes
Number of elective hernia repairs (<5 y/o) last year	Introduced by Expert Panel	0.88	N/A	Yes
Number of elective hernia repairs (5–15 y/o) last year	Introduced by Expert Panel	0.88	N/A	Yes
Number of neonatal (<1 month age) stomas performed last year	Retained from CSAT	0.82	N/A	Yes
Number of surgical repairs of pediatric (<5 y/o) open fractures performed last year	Introduced by Expert Panel	0.82	N/A	Yes
Number of surgical repairs of pediatric (5–15 y/o) open fractures performed last year	Modified	0.82	N/A	Yes
Total number of procedures performed in pediatric patients (<5 years) last year	Introduced by Expert Panel	0.88	N/A	Yes
Total number of procedures performed in pediatric patients (5–15 years) last year	Retained from CSAT	0.88	N/A	Yes
Percent of < 5 surgery cases that were emergent or urgent (non-elective) cases	Introduced by Expert Panel	0.94	N/A	Yes
Percent of 5–15 surgery cases that were emergent or urgent (non-elective) cases	Retained from CSAT	0.82	N/A	Yes
At what age do you start performing elective hydrocele/hernia repair?	Introduced by Expert Panel	0.76	N/A	Yes
Is your institution involved in a formal training program for surgical trainees?	Retained from CSAT	0.65	0.76	Yes
Does your institution have a method of monitoring surgical outcomes over time?	Retained from CSAT	0.82	N/A	Yes
Does your institution use electronic medical records?	Retained from CSAT	0.69	0.76	Yes
Does your institution have a trauma registry that includes pediatric trauma?	Retained from CSAT	0.76	N/A	Yes
Number of post-operative pediatric (<5 y/o) in-hospital deaths last year	Introduced by Expert Panel	0.88	N/A	Yes
Number of post-operative pediatric (5–15 y/o) in-hospital deaths last year	Retained from CSAT	0.82	N/A	Yes
Number of surgical site infections(SSIs) in pediatric patients (<15 y/o) in the last year ssi_all	Retained from CSAT	0.82	N/A	Yes
What is the highest ASA class of children operated at your institution? (ASA I = healthy patient, ASA II = patient with mild systemic disease, ASA III = patient with severe systemic disease, ASA IV = patient with severe systemic disease that is a constant threat to life)	Modified	0.82	N/A	Yes
Is the WHO Surgical Safety Checklist used in the operating rooms for pediatric patients?	Retained from CSAT	0.94	N/A	Yes
Is pulse oximetry used in the operating rooms for pediatric patients?	Retained from CSAT	1.00	N/A	Yes
How often does the hospital hold a surgical audit (Mortality and Morbidity conference) related to children’s surgical patients?	Retained from CSAT	0.82	N/A	Yes
Functional anesthesia machines with pediatric breathing system	Modified	0.82	N/A	Yes
Pediatric oropharyngeal airway (000–4)	Retained from CSAT	0.88	N/A	Yes
Pediatric endotracheal tubes (2.5–6 mm)	Retained from CSAT	0.93	N/A	Yes
Pediatric laryngoscope(Miller ≤2 or Macintosh ≤ 3)	Retained from CSAT	0.87	N/A	Yes
Pediatric facemask bag valve or Ambu bag (<550 ml bag with < size 3 mask)	Modified	0.94	N/A	Yes
Pediatric difficult airway kit (LMA)	Retained from CSAT	0.94	N/A	Yes
Pediatric Magill forcep	Retained from CSAT	0.88	N/A	Yes
Pediatric blood pressure monitor or cuff	Retained from CSAT	0.94	N/A	Yes
Pediatric pulse oximetry	Retained from CSAT	1.00	N/A	Yes
Pediatric nasogastric Tube (<12 Fr)	Retained from CSAT	0.94	N/A	Yes
Pediatric chest tubes (<20 Fr)	Retained from CSAT	0.94	N/A	Yes
Pediatric surgical instruments	Retained from CSAT	0.81	N/A	Yes
Pediatric urinary catheters (<12 Fr)	Retained from CSAT	1.00	N/A	Yes
Pediatric central lines (<7 Fr or 12 cm)	Retained from CSAT	0.81	N/A	Yes
Sutures (3.0, 4.0, 5.0, 6.0)	Modified	0.88	N/A	Yes
Minimally invasive equipment (laparoscopy, arthroscopy, and thoracoscopy)	Retained from CSAT	0.69	0.47	No
Capnography (exp. CO2 measurement)	Retained from CSAT	0.75	N/A	Yes
Pre-formed intestinal silos or pre-formed/readily available silos created from plastic bags	Modified	0.67	0.69	Yes
**d. WORKFORCE**				
Number of qualified general surgeons with pediatric exposure (trained surgeons with expertise in pediatric or neonatal surgery, but without formal subspecialty pediatric surgical training)	Retained from CSAT	0.88	N/A	Yes
Number of qualified general pediatric surgeons (trained surgeons with formal specialized training in children’s surgery of ≥1 year)	Retained from CSAT	0.82	N/A	Yes
Number of general doctors providing pediatric surgery (general practitioners without formal surgical training)	Retained from CSAT	0.82	N/A	Yes
Number of non-physicians providing pediatric procedure (non-physicians health care professional who performs procedures independently without formal training in surgery). These procedures include: circumcision, reduction of closed fractures, small wound repair, debridement, minor skin grafts.	Retained from CSAT	0.76	N/A	Yes
Number of qualified pediatric anesthesiologists (trained anesthesiologists with formal specialization in pediatric anesthesia of ≥1 year)	Retained from CSAT	0.88	N/A	Yes
Number of general doctors providing pediatric anesthesia (general practitioners without formal anesthesiology training)	Retained from CSAT	0.65	0.80	Yes
Number of non-physicians providing pediatric anesthesia (non-physicians health care professionals who perform pediatric anesthesia independently without formal training in anesthesia)	Retained from CSAT	0.88	N/A	Yes
Number of nurses with training or exposure in pediatric surgery, who treat only children	Retained from CSAT	0.82	N/A	Yes
Number of visiting or consultant physicians who are involved in pediatric surgical care (visiting at least 1×/week for at least 1 year).	Introduced by Expert Panel	0.71	0.88	Yes
Please identify pediatric specialists that are available at your hospital: General Pediatrician	Retained from CSAT	1.00	N/A	Yes
Please identify pediatric specialists that are available at your hospital: Cardiac surgeon	Retained from CSAT	0.53	0.59	Yes
Please identify pediatric specialists that are available at your hospital: Dental surgeon	Retained from CSAT	0.76	N/A	Yes
Please identify pediatric specialists that are available at your hospital: Neurosurgeon	Retained from CSAT	0.63	0.65	Yes
Please identify pediatric specialists that are available at your hospital: Ophthamologist	Retained from CSAT	0.71	0.65	Yes
Please identify pediatric specialists that are available at your hospital: Orthopedic surgeon	Retained from CSAT	0.82	N/A	Yes
Please identify pediatric specialists that are available at your hospital: Otorhinolaryngologist (ENT)	Retained from CSAT	0.76	N/A	Yes
Please identify pediatric specialists that are available at your hospital: Plastic surgeon	Retained from CSAT	0.76	N/A	Yes
Please identify pediatric specialists that are available at your hospital: Urologist	Retained from CSAT	0.71	0.65	Yes
Please identify pediatric specialists that are available at your hospital: Hematologist	Retained from CSAT	0.69	0.47	No
Please identify pediatric specialists that are available at your hospital: Nephrologist	Retained from CSAT	0.65	0.47	No
Please identify pediatric specialists that are available at your hospital: Neurologist	Retained from CSAT	0.63	0.53	Yes
Please identify pediatric specialists that are available at your hospital: Respiratory Physician	Retained from CSAT	0.71	0.65	Yes
Please identify pediatric specialists that are available at your hospital: Neonatologist	Retained from CSAT	0.82	N/A	Yes
Please identify pediatric specialists that are available at your hospital: Cardiologist	Retained from CSAT	0.71	0.71	Yes
Please identify pediatric specialists that are available at your hospital: Endocrinologist	Retained from CSAT	0.65	0.65	Yes
Please identify staff members that are available at your hospital: Oncologist treating children	Retained from CSAT	0.76	N/A	Yes
Please identify pediatric specialists that are available at your hospital: Physiotherapist	Introduced by Expert Panel	0.65	0.76	Yes
Please identify staff members that are available at your hospital: Pediatric intensive care nurse	Retained from CSAT	0.88	N/A	Yes
Please identify staff members that are available at your hospital: Neonatal nurse	Retained from CSAT	0.81	N/A	Yes
Please identify staff members that are available at your hospital: Qualified nutritionist	Retained from CSAT	0.76	N/A	Yes
Please identify staff members that are available at your hospital: Radiographer	Retained from CSAT	0.82	N/A	Yes
Please identify staff members that are available at your hospital: Radiologist	Retained from CSAT	0.82	N/A	Yes
Please identify staff members that are available at your hospital: Speech therapist	Introduced by Expert Panel	0.65	0.65	Yes
Please identify staff members that are available at your hospital: Audiometrist	Retained from CSAT	0.53	0.65	Yes
Please identify staff members that are available at your hospital: Pathologist	Retained from CSAT	0.71	0.71	Yes
Please identify staff members that are available at your hospital: Ophthalmology technician	Introduced by Expert Panel	0.71	0.75	Yes
How often are these available 24 hours a day?: General surgeon or pediatric surgeon	Retained from CSAT	0.82	N/A	Yes
How often are these available 24 hours a day?: Pediatric anesthesia provider availability (with >6 months of training or exposure in pediatrics)	Retained from CSAT	0.88	N/A	Yes
**e. FINANCING**				
What percentage of children coming to this hospital have health insurance	Retained from CSAT	0.88	N/A	Yes
Nationally, is there government-sponsored health insurance/financing for children?	Retained from CSAT	0.82	N/A	Yes
On average, what percentage pediatric surgical costs for patients are covered by insurance?	Retained from CSAT	0.88	N/A	Yes
Annual hospital budget allotted to children’s surgery and anesthesia	Retained from CSAT	0.88	N/A	Yes
Average total inpatient cost for a patient for: pediatric hernia repair	Retained from CSAT	0.71	0.82	Yes
Average total inpatient cost for a patient for: pediatric open fracture repair	Retained from CSAT	0.71	0.82	Yes
Average total inpatient cost for a patient for: pediatric laparotomy	Retained from CSAT	0.76	N/A	Yes
Average total inpatient cost for a patient for: Pediatric appendectomy	Introduced by Expert Panel	0.76	N/A	Yes
Average total inpatient cost for a patient for: repair of Hirschprung’s disease/anorectal malformation	Removed	N/A	N/A	No
Average non-medical/additional cost to a patient for a pediatric appendectomy (transport, food/lodging, missed work for family members)	Introduced by Expert Panel	0.76	N/A	Yes
**f. TRAINING AND RESEARCH**				
How many ongoing research projects involve children’s surgery (this may include quality improvement projects)?	Modified	0.71	0.88	Yes
How many ongoing research projects involve pediatric anesthesia?	Retained from CSAT	0.71	0.82	Yes
How many ongoing research projects involve pediatric nursing?	Retained from CSAT	0.71	0.88	Yes
How many workshops, trainings, and lectures related to pediatric surgery or perioperative care are in an average month?	Retained from CSAT	0.88	N/A	Yes

*Items that scored 0.75 or above in Round 2 were retained and not further discussed in Round 3. These are listed as N/A below.

### Round 2

Seventeen of the (94%) expert panellists participated in the second-round survey. Of the 181 items included in the second-round CSAT adaptation, 43 items that did not meet the threshold criteria of 0.75 consensuses for inclusion.

### Round 3

Seventeen (94%) expert panellists participated in the third round. Out of the 43 items evaluated in the third round, there were 10 items that did not meet a 50% agreement for inclusion on the survey and were therefore removed from the final survey adaptation.

### CVI

The I-CVI ranged from 0.53 for ‘Does your hospital have a paediatric neurologist’ to 0.94 (nine items: total number of paediatric inpatient admissions for children <5 in a year, total number of children’s surgical admissions for children <5 in a year, total number of paediatric ED visits for children 5–15 years-old in a year, total number of functional incubators or paediatric warmers, most common reason for referral of paediatric surgical patients 5–15 years-old, how far away is the nearest referral hospital (hours, by car), does your hospital have an anaesthesiologist who can perform sedation, per cent surgery cases that were emergent or urgent (non-elective) cases for children of <5 years-old, and is the WHO Surgical Safety Checklist used in the operating rooms for paediatric patients). A full list of I-CVI scores for each item can be found in Table 2. The average S-CVI for the 171 items included on the final survey was 0.84 (standard deviation = .10).

## Discussion

Scaling up basic surgical services at district hospitals has the potential to avert significant morbidity and mortality for children in LMICs. However, there is not yet a context-specific comprehensive tool to evaluate current capacity at the district hospital level. The aim of this study was to develop a context-specific paediatric surgery assessment tool for district hospitals in Rwanda. The main adaptations of the original CSAT relate to evaluating access for different age ranges (<5 and 5–15) and ensuring that the evaluated procedures are appropriate for district hospitals. Compared to the original CSAT, our Rwandan CSAT (CSAT_R_) introduced 42 new items, modified 19 items, and retained 112 items as they were (final tool can be found in Supplemental Table S1).

When compared to existing paediatric surgical assessment tools such as Pedi-PIPES or paediatric Bellwether procedures, our CSAT_R_ stands out as a more comprehensive tool. It includes items related to training and education, as well as an adequate focus on neonatal conditions. However, the original CSAT may overlook specific barriers and gaps that are unique to the context of district hospitals in East Africa. Our tool is specifically tailored to district hospitals and considers their distinct requirements and limitations. For example, we chose to further differentiate surgical care provision by age. In Rwanda, one of the main barriers to providing surgical care for children under 5 years-old is a lack of a physician anaesthesiologist. Non-physician anaesthetists are often the primary anaesthesia providers, particularly in district hospital settings. Given that national regulations require physician anaesthesiologists to provide general anaesthesia for patients under 5 years-old, our expert panel felt that it was important to evaluate barriers to surgical care for these two paediatric age groups separately. Moreover, our tool complements the Adult SAT, enabling district hospital s to conduct a comprehensive baseline capacity assessment for providing surgical care.

This study represents the first adaptation and validation of the CSAT for a district hospital setting. A notable strength of our study lies in the robustness of the expert panel involved in developing the tool, with over 70% of panel members from Rwanda, ensuring local expertise and knowledge were incorporated. However, it is important to acknowledge that our tool’s specific adaptation for district hospital s in Rwanda may limit its generalisability to other LMICs or different district hospital settings. Nevertheless, given the incorporation of both local and global health systems-level experts, we believe our tool can serve as a foundation for other district hospital s in East Africa. Future studies can follow a similar methodology to adapt the tool to fit their own contexts as needed.

One limitation of the study was conflicting opinions regarding what aspects were essential to assess, considering the balance between tool length and comprehensiveness. Additionally, the relevance of including certain areas in the assessment tool, such as emergency department visits, intensive care units, and pathology services should be carefully considered, focusing only on components pertinent to the district hospital setting. Evaluating both specialists and non-specialist care providers, such as non-physician anaesthetists and operating room nurses who often treat paediatric patients in district hospitals may provide valuable insights into paediatric surgical care. During the implementation of the tool, we received feedback from data collectors, suggesting the incorporation of branch logic to exclude irrelevant items in settings where certain information is not routinely collected by district hospitals. These insights gained during implementation should be considered in future iterations of the tool.

Finally, it is also important to acknowledge the potential for error resulting from expert panellists’ providing feedback based on what they perceive as important for paediatric surgical care versus what they believe should be included on the tool (i.e. evaluated at a district hospital level). While efforts were made to clarify this point with each individual expert panellist, future implementations of the tool should provide clear instructions to avoid confusion and enhance the accuracy of the collected data.

In conclusion, this study successfully developed a context-specific paediatric surgery assessment tool for district hospitals in Rwanda. The tool can be expanded for use in other district hospitals, aiming to increase access to basic and essential paediatric surgery capacity appropriate for a first-level district hospital. This is particularly important to enhance access to care for children at the local level and reduce the backlog of cases referred to tertiary centres, enabling them to focus on more emergent and higher complexity cases. The adapted tool can serve as a starting point for other district hospital s in LMICs, particularly in neighbouring countries in East Africa. The methodology employed in this study can be mirrored in other studies, tailoring it to their specific contexts and contributing to the overall improvement of paediatric surgical care in district hospitals.

## Supplementary Material

Supplemental Table 1_Global Health Action_CSATR Final.docx

## Paper context

To date, there are limited tools to assess paediatric surgery capacity in LMICs. This study adapted and validated the Children’s Surgical Assessment Tool (CSAT), a tool developed for district and tertiary hospitals in Nigeria to evaluate hospital infrastructure, workforce, service delivery, financing, and training capacity for paediatric surgery, for use in district hospitals in Rwanda.
