# Supplementary material for: Adaptation and validation of the Children’s Surgical Assessment Tool for Rwandan district hospitals
Source: Glob Health Action. 2024 Jan 9;17(1):2297870. doi: 10.1080/16549716.2023.2297870 (PMC10778412; doi:10.1080/16549716.2023.2297870)
Supplement: Supplemental Table 1_Global Health Action_CSATR Final.docx [file ZGHA_A_2297870_SM9211.docx]

**Children’s Surgical Assessment Tool: For Rwanda District Hospitals**

Comments

| **FACILITY CHARACTERISTICS** | | |
| --- | --- | --- |
| **For children <5 years old:** | | |
|  | Total number of pediatric inpatient admissions (>6 hours in non-emergency ward) for children <5 in a year | # |
|  | Total number of pediatric Emergency Department visits for children <5 in a year | # |
|  | Total number of pediatric outpatient visits for children <5 in a year | # |
|  | Total number of children’s surgical admissions for children <5 in a year | # |
|  | Total number of children’s surgical outpatients (visits) for children < 5 seen in a year | # |
| **For children 5-15 years old:** | | |
|  | Total number of pediatric inpatient admissions (>6 hours in non-emergency ward) for children 5-15 years-old in a year | # |
|  | Total number of pediatric Emergency Department visits for children 5-15 years-old in a year | # |
|  | Total number of pediatric outpatient visits for children 5-15 years-old in a year | # |
|  | Total number of children’s surgical admissions for children 5-15 years-old in a year | # |
|  | Total number of children’s surgical outpatients (visits) for children 5-15 years-old seen in a year | # |
| **Facility Information:** | | |
|  | Total number of inpatient hospital beds dedicated to children’s surgery | # |
|  | Does your hospital have a pediatric recovery room? | Y or N |
|  | Total number of recovery room hospital beds dedicated to children’s surgery | # or N/A |
|  | Is oxygen available in the recovery room? | Y or N or N/A |
|  | Total number of operating rooms in the hospital | # |
|  | Total number children's operating rooms | # |
|  | Total number of neonatal unit/special baby care unit beds | # |
|  | Does your hospital have a post-operative ICU? |  |
|  | Total number of pediatric ICU/advanced care beds | # |
|  | Total number of neonatal ICU beds | # |
|  | Total number of functional pediatric ventilators in the ICU | # or N/A |
|  | Total number of functional neonatal ventilators in ICU | # or N/A |
|  | Total number of functional incubators or pediatric warmers | # |
|  | Total number of patient monitors in pediatric and neonatal ICU | # or N/A |
|  | How many of these monitors include all of the following: EKG, blood pressure, pulse oximetry, temperature and capnography? | # |
| **Access and referral systems:** | |  |
|  | How many patients <5 years old per year do you refer to higher-level facilities for surgical interventions? | # |
|  | How many patients 5-15 years old per year do you refer to higher-level facilities for surgical interventions? | # |
|  | What is the most common reason for referral of pediatric surgical patients < 5 years old to higher levels of care? |  |
|  | What is the most common reason for referral of pediatric surgical patients 5-15 years old to higher levels of care? |  |
|  | How far away is the nearest referral hospital (hours, by car) | ___ hours |

Comments

Comments

| **INFRASTRUCTURE** | | | | | | |
| --- | --- | --- | --- | --- | --- | --- |
| General Infrastructure - How often is this item available and functional? Choose (tick) 0- Unavailable (NOT AVAILABLE under any circumstances); 1- Inadequate (available to LESS THAN HALF of the time); 2- Limited (available to MORE THAN HALF, of the time but not all of the time); or 3- Adequate (AVAILABLE all of the time without restrictions). | | Unavailable (0) | Inadequate (1) | | Limited (2) | Adequate (3) |
|  | 24-hour Emergency Unit able to receive pediatric patients |  |  |  | |  |
| Pharmacy – product availability | | | | | | |
|  | Pediatric malnutrition feeding program |  |  |  | |  |
|  | Pediatric dosing cognitive aid or guide |  |  |  | |  |
| Radiology & Pathology – service availability | | | | | | |
|  | Does your hospital have a radiologist? | Y or N | | | | |
|  | Does your hospital have an anesthesiologist who can perform sedation? | Y or N | | | | |
|  | Echocardiogram |  |  |  | |  |
|  | Ultrasound |  |  |  | |  |
|  | X-ray |  |  |  | |  |
|  | Anatomic Pathology services |  |  |  | |  |
| Blood Supply - availability | | | | | | |
|  | Blood component transfusion |  |  |  | |  |

| **SERVICE DELIVERY** |
| --- |
| **In column 1 (procedure performed), indicate if the procedure is performed:**  Yes in all pediatric patients < 15  Yes, but not in under 5 patients  Yes, depending on complexity  Yes, in emergency only or as temporizing measure   1. No, never   **In column 3, Rate adequacy as below:** 0- Unavailable (NOT AVAILABLE under any circumstances); 1- Inadequate (available to LESS THAN HALF of the time); 2- Limited (available to MORE THAN HALF, of the time but not all of the time) 3- Adequate (AVAILABLE all of the time without restrictions).  **Barriers:** If Unavailable, Inadequate or Limited, (<3 in Adequacy column) please identify the barriers to access (check all that apply):  Infrastructure - physical space, equipment or materials;  Absent - has never has been present;  Broken –resources present, but broken;  Personnel - resource, service or function available, and staff trained, but limited availability at times (eg, night, weekend or holiday);  Training – No staff trained in using resource or performing function;  Stock out - cannot be procured, or required equipment or supplies out of stock often due to poor stock management practices or procurement failures;  User fees - available, but out-of-pocket payment requirement prevents delivery for some;  Other - Other factors (if “other,” please indicate in the box by writing the letter of which of the following best corresponds to the barrier).   - (A) Lack of anesthesiologist or general anesthesia - (B) Lack of specialist (orthopedist, eye clinic etc) - (C) Lack of imaging or pathology services - (D) Other not specified (please list in comments) |

| **SERVICE DELIVERY CONTINUED**  Comments | | | | | | | | | | | | | | | | | |
| --- | --- | --- | --- | --- | --- | --- | --- | --- | --- | --- | --- | --- | --- | --- | --- | --- | --- |
|  | | **Procedure Performed** | **Total # performed/year** | | **Rate (0-3)** | | **Infrastructure** | | **Absent** | **Broken** | **Personnel** | **Training** | **Stock Out** | | **User Fees** | | **Other** |
| **Procedures- Minor in patients <15 years old** | | | | | | | | | | | | | | | | |  |
|  | Suturing laceration |  |  |  | |  | |  | |  |  |  |  |  | |  | |
|  | Drainage of superficial abscess |  |  |  | |  | |  | |  |  |  |  |  | |  | |
|  | Wound debridement |  |  |  | |  | |  | |  |  |  |  |  | |  | |
|  | Biopsy (tumor) |  |  |  | |  | |  | |  |  |  |  |  | |  | |
|  | Male circumcision |  |  |  | |  | |  | |  |  |  |  |  | |  | |
|  | Management of non-displaced fractures |  |  |  | |  | |  | |  |  |  |  |  | |  | |
|  | Removal of foreign body from ear/nose |  |  |  | |  | |  | |  |  |  |  |  | |  | |
|  | Support for emergency airway obstruction |  |  |  | |  | |  | |  |  |  |  |  | |  | |
|  | Reduction of dislocation |  |  |  | |  | |  | |  |  |  |  |  | |  | |
|  | Reduction and application of splint for non-displaced fractures |  |  |  | |  | |  | |  |  |  |  |  | |  | |
|  | IV placement for neonates |  |  |  | |  | |  | |  |  |  |  |  | |  | |
| **Procedures – Major in patients < 15 years old** | | | | | | | | | | | | | | | | | |
| *Children’s surgery* | | | | | | | | | | | | | | | | | |
|  | Appendectomy |  |  |  | |  | |  | |  |  |  |  |  | |  | |
|  | Hernia/hydrocele repair |  |  |  | |  | |  | |  |  |  |  |  | |  | |
|  | Non-operative reduction of intussusception |  |  |  | |  | |  | |  |  |  |  |  | |  | |
|  | Operative reduction of intussusception |  |  |  | |  | |  | |  |  |  |  |  | |  | |
|  | Bowel resection |  |  |  | |  | |  | |  |  |  |  |  | |  | |
|  | Temporizing measures for gastroschisis and omphalocele (cover and rehydration) prior to referral |  |  |  | |  | |  | |  |  |  |  |  | |  | |
|  | Rectal biopsy |  |  |  | |  | |  | |  |  |  |  |  | |  | |
|  | Resection of solid abdominal masses |  |  |  | |  | |  | |  |  |  |  |  | |  | |
|  | Creation of intestinal stomas |  |  |  | |  | |  | |  |  |  |  |  | |  | |
|  | Emergency ostomies for imperforate anus |  |  |  | |  | |  | |  |  |  |  |  | |  | |
|  | Closures of intestinal stomas |  |  |  | |  | |  | |  |  |  |  |  | |  | |
|  | Resuscitation for pyloric stenosis |  |  |  | |  | |  | |  |  |  |  |  | |  | |
|  | Catheterization / suprapubic cystostomy |  |  |  | |  | |  | |  |  |  |  |  | |  | |
|  | Orchiopexy |  |  |  | |  | |  | |  |  |  |  |  | |  | |
|  | Repair of testicular or ovarian torsion |  |  |  | |  | |  | |  |  |  |  |  | |  | |
|  | Drainage of septic arthritis / osteomyelitis |  |  |  | |  | |  | |  |  |  |  |  | |  | |
|  | Repair of cleft lip and/or palate |  |  |  | |  | |  | |  |  |  |  |  | |  | |
| *Pediatric Resuscitation and Injury* | | | | | | | | | | | | | | | | | |
|  | Emergency Surgical airway (cricothyroidotomy) |  |  |  | |  | |  | |  |  |  |  |  | |  | |
|  | Emergency Tube thoracostomy |  |  |  | |  | |  | |  |  |  |  |  | |  | |
|  | Trauma Laparotomy |  |  |  | |  | |  | |  |  |  |  |  | |  | |
|  | Conservative (non-operative) management for simple humeral fracture |  |  |  | |  | |  | |  |  |  |  |  | |  | |
|  | Open reduction and internal fixation |  |  |  | |  | |  | |  |  |  |  |  | |  | |
|  | Placement of pediatric external fixator |  |  |  | |  | |  | |  |  |  |  |  | |  | |
|  | Emergency Escharotomy/fasciotomy |  |  |  | |  | |  | |  |  |  |  |  | |  | |
|  | Contracture release |  |  |  | |  | |  | |  |  |  |  |  | |  | |
|  | Amputations |  |  |  | |  | |  | |  |  |  |  |  | |  | |
|  | Skin grafting |  |  |  | |  | |  | |  |  |  |  |  | |  | |
|  | Emergency Burr hole |  |  |  | |  | |  | |  |  |  |  |  | |  | |
|  | Emergency Craniotomy |  |  |  | |  | |  | |  |  |  |  |  | |  | |
| **Procedures - Advanced (<15 years old)** | | | | | | | | | | | | | | | | | |
|  | Surgery for neonatal acute abdomen |  |  |  | |  | |  | |  |  |  |  |  | |  | |
|  | Repair of club foot |  |  |  | |  | |  | |  |  |  |  |  | |  | |

| **Surgical Volume** | | |
| --- | --- | --- |
|  | Number of pediatric laparotomies (<5 y/o) performed last year | # |
|  | Number of pediatric laparotomies (5-15 y/o) performed last year | # |
|  | Number of elective hernia repairs (<5 y/o) last year | # |
|  | Number of elective hernia repairs (5-15 y/o) last year | # |
|  | Number of neonatal (< 1 month age) stomas performed last year | # |
|  | Number of surgical repairs of pediatric (<5 y/o) open fractures performed last year | # |
|  | Number of surgical repairs of pediatric (5-15 y/o) open fractures performed last year | # |
|  | Total number of procedures performed in pediatric patients (<5 years) last year | # |
|  | Total number of procedures performed in pediatric patients (5-15 years) last year | # |
|  | Percent of <5 surgery cases that were emergent or urgent (non-elective) cases | % |
|  | Percent of 5-15 surgery cases that were emergent or urgent (non-elective) cases | % |
|  | At what age do you start performing elective hydrocele/hernia repair? | ____ Years |

Comments

| **Quality and Safety** | | |
| --- | --- | --- |
|  | Is your institution involved in a formal training program for surgical trainees? |  Yes  No |
|  | Does your institution have a method of monitoring surgical outcomes over time? |  Yes  No |
|  | Does your institution use electronic medical records? |  Yes  No |
|  | Does your institution have a trauma registry that includes pediatric trauma? |  Yes  No |
|  | Number of post-operative pediatric (<5 y/o) in-hospital deaths last year | # |
|  | Number of post-operative pediatric (5-15 y/o) in-hospital deaths last year | # |
|  | Number of surgical site infections(SSIs) in pediatric patients (< 15 y/o) in the last year | # |
|  | What is the highest ASA class of children operated at your institution? (ASA I = healthy patient, ASA II = patient with mild systemic disease, ASA III = patient with severe systemic disease, ASA IV = patient with severe systemic disease that is a constant threat to life) | 1 2 3 4 |
|  | Is the WHO Surgical Safety Checklist used in the operating rooms for pediatric patients? |  Never  Less than half the time  More than half the time  All the time |
|  | Is pulse oximetry used in the operating rooms for pediatric patients? |  Never  Less than half the time  More than half the time  All the time |
|  | How often does the hospital hold a surgical audit (Mortality and Morbidity conference) related to children’s surgical patients? |  Never  Every month  As needed _______________   Every week  Every quarter  Other |

Comments

| **Operating Equipment and Supplies – How often is the following equipment available and functional for surgery?** | | | | | | | | | | | | |
| --- | --- | --- | --- | --- | --- | --- | --- | --- | --- | --- | --- | --- |
|  |  | | **Rate (0-3)** | | **Infrastructure** | **Absent** | **Broken** | **Personnel** | **Training** | **Stock Out** | **User Fees** | **Other** |
|  | | Functional anesthesia machines with pediatric breathing system | |  |  |  |  |  |  |  |  |  |
|  | | Pediatric oropharyngeal airway *(000-4)* | |  |  |  |  |  |  |  |  |  |
|  | | Pediatric endotracheal tubes *(2.5 - 6 mm)* | |  |  |  |  |  |  |  |  |  |
|  | | Pediatric laryngoscope*(Miller ≤ 2 or Macintosh ≤ 3)* | |  |  |  |  |  |  |  |  |  |
|  | | Pediatric facemask bag valve or Ambu bag *(< 550ml bag with < size 3 mask)* | |  |  |  |  |  |  |  |  |  |
|  | | Pediatric difficult airway kit (LMA) | |  |  |  |  |  |  |  |  |  |
|  | | Pediatric Magill forceps | |  |  |  |  |  |  |  |  |  |
|  | | Pediatric blood pressure monitor or cuff | |  |  |  |  |  |  |  |  |  |
|  | | Pediatric pulse oximetry | |  |  |  |  |  |  |  |  |  |
|  | | Pediatric nasogastric Tube *( <12 Fr)* | |  |  |  |  |  |  |  |  |  |
|  | | Pediatric chest tubes *(< 20 Fr)* | |  |  |  |  |  |  |  |  |  |
|  | | Pediatric surgical instruments | |  |  |  |  |  |  |  |  |  |
|  | | Pediatric urinary catheters *(<12 Fr)* | |  |  |  |  |  |  |  |  |  |
|  | | Pediatric central lines *(< 7 Fr or 12 cm* | |  |  |  |  |  |  |  |  |  |
|  | | Sutures *(3.0, 4.0, 5.0, 6.0)* | |  |  |  |  |  |  |  |  |  |
|  | | Capnography (exp. CO2 measurement) | |  |  |  |  |  |  |  |  |  |
|  | | Pre-formed intestinal silos or pre-formed/readily available silos created from plastic bags | |  |  |  |  |  |  |  |  |  |

Comments

| **WORKFORCE** | | | | | | | | | |
| --- | --- | --- | --- | --- | --- | --- | --- | --- | --- |
| **Children's Surgery Provider Density**  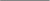 | | | | | | | | | |
| **Providers** | | | | | **Full Time** | | | **Part Time** | |
|  | Number of qualified general surgeons with pediatric exposure (trained surgeons with expertise in pediatric or neonatal surgery, but without formal subspecialty pediatric surgical training) | | | | # | | | # | |
|  | Number of qualified general pediatric surgeons (trained surgeons with formal specialized training in children’s surgery of ≥ 1 year) | | | | # | | | # | |
|  | Number of general doctors providing pediatric surgery (general practitioners without formal surgical training) | | | | # | | | # | |
|  | Number of non-physicians providing pediatric procedure (non-physicians health care professional who performs procedures independently without formal training in surgery). These procedures include: circumcision, reduction of closed fractures, small wound repair, debridement, minor skin grafts. | | | | # | | | # | |
|  | Number of qualified anesthesiologists | | | | # | | | # | |
|  | Number of qualified pediatric anesthesiologists (trained anesthesiologists with formal specialization) | | | | # | | | # | |
|  | Number of general doctors providing pediatric anesthesia (general practitioners without formal anesthesiology training) | | | | # | | | # | |
|  | Number of non-physicians providing pediatric anesthesia (non-physicians health care professionals who perform pediatric anesthesia independently without formal training in anesthesia) | | | | # | | | # | |
|  | Number of nurses with training or exposure in pediatric surgery, who treat only children | | | |  | | |  | |
|  | Number of visiting or consultant physicians who are involved in pediatric surgical care (visiting at least 1x/week for at least 1 year). | | | | Type _______________ # ____________  Type _______________ # ____________  Type _______________ # ____________ | | | | |
| Please identify pediatric specialists that are available at your hospital *(indicate number in brackets)* | | | | | | | | | |
| * general pediatrician ( )*  * cardiac surgeon ( )   dental surgeon ( )  neurosurgeon ( )  ophthalmologist( ) (  orthopedic surgeon( )  otorhinolaryngologist (ENT) ( )*  * plastic surgeon ( )*  * urologist ( )* | | | * neurologist ( )  respiratory physician ( )*  * neonatologist ( )*  * cardiologist ( )*  * endocrinologist ( )*  * physiotherapist ( )* | | | | | | |
| Please identify staff members that are present in your hospital (indicate number in brackets) | | | | | | | | | |
|  pediatric intensive care nurse ( )   neonatal nurse ( )   qualified nutritionist ( ) | |  radiographer ( )   radiologist ( )   speech therapist ( )   audiologist ( ) | | | |  pathologist ( )   oncologist treating children ( )   ophthalmology technician ( ) | | | |
|  |  | | | | | | | | |
| **Work Force Availability (How often are these available 24 hours a day?)** | | | | Unavailable (0) | | | Inadequate (1) | Limited (2) | Adequate (3) |
|  | General surgeon or pediatric surgeon availability | | |  | | |  |  |  |
|  | Pediatric anesthesia provider availability (with >6 months of training or exposure in pediatrics) | | |  | | |  |  |  |

Comments

| **FINANCING** | | | |
| --- | --- | --- | --- |
| **Health financing and accounting** | | | |
|  | What percentage of children coming to this hospital have health insurance |  None  Fewer than half  More than half  All | |
|  | Nationally, is there government-sponsored health insurance/financing for children? |  Yes  No | |
|  | On average, what percentage pediatric surgical costs for patients are covered by insurance? | % | |
| **Budget Allocation** | | | |
|  | Annual hospital budget allotted to children’s surgery and anesthesia  N/A | Actual amount: % of total budget: | |
| **Cost: average total inpatient cost for a patient for…** | | Cost | % Out of Pocket |
|  | pediatric hernia/hydrocele repair |  |  |
|  | pediatric fracture management |  |  |
|  | pediatric laparotomy |  |  |
|  | Pediatric appendectomy |  |  |
| **Average non-medical cost to a patient for a pediatric hernia/hydrocele repair** (consider transport, lodging, food for patient and family) | | Cost | |
|  | |  | |

Comments

| **TRAINING AND RESEARCH** | | |
| --- | --- | --- |
|  | How many ongoing research projects involve children’s surgery (this may include quality improvement projects)? | # |
|  | How many ongoing research projects involve pediatric anesthesia? | # |
|  | How many ongoing research projects involve pediatric nursing? | # |
|  | How many workshops, trainings, and lectures related to pediatric surgery or perioperative care are in an average month? | # |
